# The Effect of 10 Hz Repetitive Transcranial Magnetic Stimulation of Posterior Parietal Cortex on Visual Attention

**DOI:** 10.1371/journal.pone.0126802

**Published:** 2015-05-13

**Authors:** Isabel Dombrowe, Georgiana Juravle, Mohsen Alavash, Carsten Gießing, Claus C. Hilgetag

**Affiliations:** 1 Department of Experimental Psychology, Otto-von-Guericke-University Magdeburg, Magdeburg, Germany; 2 Department of Computational Neuroscience, University Medical Center Hamburg-Eppendorf, Hamburg, Germany; 3 Department of Systems Neuroscience, University Medical Center Hamburg-Eppendorf, Hamburg, Germany; 4 Biological Psychology Lab, Department of Psychology, European Medical School, University of Oldenburg, Oldenburg, Germany; 5 Department of Health Sciences, Boston University, Boston, USA; Università di Trento, ITALY

## Abstract

Repetitive transcranial magnetic stimulation (rTMS) of the posterior parietal cortex (PPC) at frequencies lower than 5 Hz transiently inhibits the stimulated area. In healthy participants, such a protocol can induce a transient attentional bias to the visual hemifield ipsilateral to the stimulated hemisphere. This bias might be due to a relatively less active stimulated hemisphere and a relatively more active unstimulated hemisphere. In a previous study, Jin and Hilgetag (2008) tried to switch the attention bias from the hemifield ipsilateral to the hemifield contralateral to the stimulated hemisphere by applying high frequency rTMS. High frequency rTMS has been shown to excite, rather than inhibit, the stimulated brain area. However, the bias to the ipsilateral hemifield was still present. The participants’ performance decreased when stimuli were presented in the hemifield contralateral to the stimulation site. In the present study we tested if this unexpected result was related to the fact that participants were passively resting during stimulation rather than performing a task. Using a fully crossed factorial design, we compared the effects of high frequency rTMS applied during a visual detection task and high frequency rTMS during passive rest on the subsequent offline performance in the same detection task. Our results were mixed. After sham stimulation, performance was better after rest than after task. After active 10 Hz rTMS, participants’ performance was overall better after task than after rest. However, this effect did not reach statistical significance. The comparison of performance after rTMS with task and performance after sham stimulation with task showed that 10 Hz stimulation significantly improved performance in the whole visual field. Thus, although we found a trend to better performance after rTMS with task than after rTMS during rest, we could not reject the hypothesis that high frequency rTMS with task and high frequency rTMS during rest equally affect performance.

## Introduction

Visual attention is controlled by a fronto-parietal attention network in the human brain [2–5]. Damage to the parietal part of this network often leads to deficits in attentional processing, such as neglect and extinction [6–8]. Whereas patients with neglect fail to attend to the visual hemifield contralateral the lesioned brain hemisphere, extinction patients are able to detect a single stimulus in one half of the visual field, but fail to detect a second, simultaneously presented stimulus in the other half of the visual field, e.g. [9, 10]. This deficit might be due to an activity imbalance between the brain hemispheres, with a relatively more active intact hemisphere and a relatively less active lesioned hemisphere [11].

Attentional deficits similar to neglect and extinction can be transiently induced in healthy participants by using repetitive transcranial magnetic stimulation (rTMS) of the posterior parietal cortex (PPC) at frequencies lower than 5 Hz [12]. These attentional biases are presumably caused by a transient inhibition of the stimulated cortical area [13]. Hilgetag, Theoret and Pascual-Leone, for example, applied rTMS for 10 minutes at a frequency of 1 Hz to the right PPC of a group of healthy participants between two successive blocks of a visual detection task [14]. They found that, after rTMS, their participants were impaired in detecting the left stimulus of a bilateral stimulus pair and showed an additional enhanced detection of stimuli presented in the right half of the visual field, an attentional imbalance similar to the one exhibited by extinction patients [14].

Further, it has been found that rTMS at 5 Hz and higher tends to excite, rather than inhibit, the cortex [15–17]. According to the imbalance-account [11], one would expect detection performance to improve for stimuli presented in the hemifield contralateral to the stimulated hemisphere and to decrease for stimuli in the ipsilateral hemifield, that is, one would expect a reversal of the results of Hilgetag and colleagues. Thus, in a follow-up study, Jin and Hilgetag [1] stimulated the same area within the PPC with a 20 Hz rTMS protocol using the same detection task as in the previous study. Contrary to their expectation, the authors obtained results that were very similar to the earlier 1 Hz study: The participants were worse at detecting stimuli presented in the visual hemifield contralateral to the stimulated hemisphere after they had received 20 Hz rTMS over the PPC, as compared to visual detection performance before stimulation.

One possibility why Jin and Hilgetag’s [1] high frequency stimulation did not lead to an improved detection performance in the contralateral hemisphere is that the rTMS was applied while participants were resting, that is, not performing a task.

Several studies have shown that the effects of TMS and rTMS depend on the behavioral context during stimulation [18–20]. Johnson and colleagues, for example, recorded the electroencephalogram (EEG) and applied single TMS pulses to the superior parietal lobule of the PPC either during the delay period of a working memory task, or during passive fixation. Their results indicated that the strength of the TMS-induced electrical currents, the spatial spread of the TMS-induced activity, as well as the TMS ability of resetting the ongoing neuronal oscillations were all greater when the TMS was applied during the memory task, as compared to when it was applied during passive fixation. With the result of Johnson and colleagues in mind, it is conceivable that the 20 Hz stimulation in the study of Jin and Hilgetag [1] increased activity only locally. Failing to activate other task relevant regions of the brain, such a protocol probably provoked a local perturbation of the stimulated area that was qualitatively similar to the perturbation caused by the 1 Hz stimulation.

The aim of the present study was to directly compare the effects of high frequency rTMS during passive rest and active task performance. To this end, we stimulated the PPC of our participants before they were performing a visual detection task either during *Rest* or while they were doing the same detection *Task* that they also performed after stimulation when performance was measured. We chose to stimulate at 10 Hz, since numerous EEG studies have shown that the alpha band frequency (8–14 Hz) is associated with attentional processing, e.g. [21–23].

The participants’ task was to detect a small Gabor patch presented 20 degrees on the left, right, or bilaterally of the fixation cross in the center of the screen.

## Materials and Methods

### Ethics statement

The study was approved by the local ethics committee (Ethik-Kommission der Äerztekammer Hamburg, no. PV4012) and was in compliance with the Declaration of Helsinki (2008) and conducted according to the TMS safety guidelines [24, 25].

### Participants

Twelve students (age range 18–35 years, 5 male) were paid for their participation. They all reported normal or corrected to normal visual acuity. All participants reported to be right handed. Before the start of the experiment, participants received written information about rTMS and signed informed consent.

### Apparatus

The experiment took place in a dimly lit room (40 *cd*/*m* ^2^). A Macbook laptop computer (2GHz CPU, 2GB RAM) generated the stimuli using the Psychophysics Toolbox 3 [26] for Matlab (Matlab 2008a, Mathworks, Natick, MA) on a 24 inch Dell monitor operating at 120Hz. Viewing distance was set to 50 cm and it was maintained during the experiment by use of a chin rest. Manual responses were collected with a standard keyboard (Dell KB1421). Participants wore earplugs (3M) for the entire duration of the experimental sessions, in order to limit the noise from the TMS device.

### Procedure

The participants performed a visual detection task. Each trial started with the presentation of a fixation cross (1.6 degrees of visual angle) in the center of the screen. Participants were asked to continuously fixate the cross. In a time window of 800 ms to 868 ms after the onset of the fixation cross, a small Gabor patch of about 2 degrees diameter briefly appeared (42 ms) approximately 20 degrees in the periphery, with equal probability either on the *left* side of fixation, on the *right* side of fixation, or *bilaterally*. Participants had to indicate whether the Gabor patch was presented on the left side, the right side, or bilaterally by pressing the left arrow key, right arrow key or down arrow key, respectively. They used their right hand to respond and were instructed to do so as fast and as accurately as possible. Gabor contrast was determined individually for each participant before the start of the experiment by using a custom staircase procedure that identified the contrast level for which the participant performed the task at an accuracy of 70% correct.

### Design

**Fig 1:**
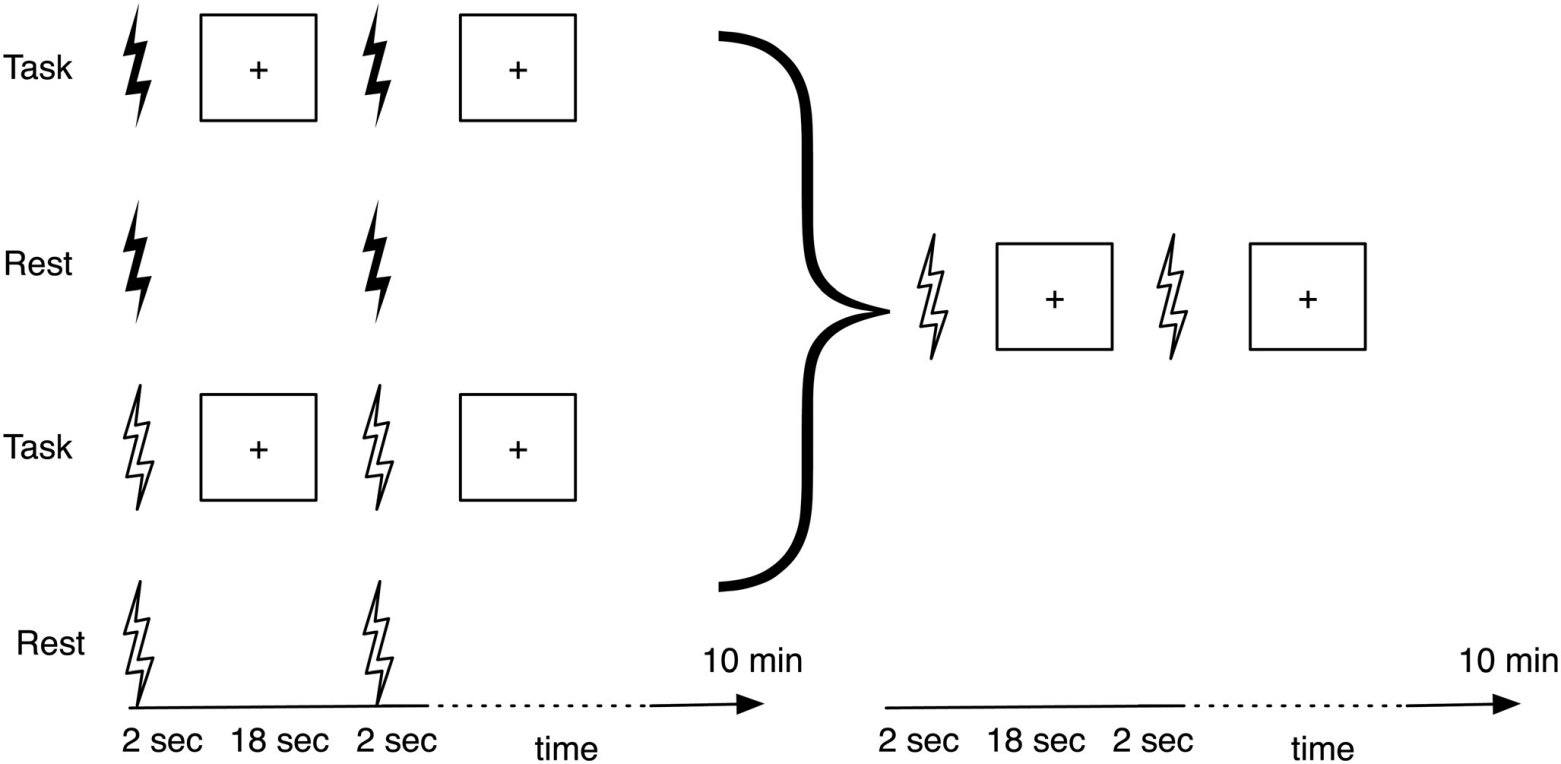
Experimental design and timeline for one experimental session of the experiment. Filled lightning signs indicate the *rTMS*, whereas the empty signs indicate the *Sham* stimulation. The screen symbols represent the occurence of the visual detection task. In the stimulation phase (first 10 min) participants received either rTMS or sham stimulation while they were performing the task or resting passively. In the subsequent test phase, participants performed the task while they were receiving sham stimulation.

The experimental design is outlined in Fig 1. We used a repeated measures, fully crossed factorial design. This design allowed us not only to assess the main effects, such as the difference between performance after task and performance after rest, but also the interactions among factors. The first factor (i.e. independent variable) was condition, with the levels of *Task* (i.e., visual detection of Gabor patches) and *Rest* (i.e., rest with eyes closed). For each of the conditions, participants received either 10 Hz *rTMS* or *Sham* (second factor, stimulation type), leading to four different combinations: *Task* with *rTMS*, *Task* with *Sham*, *Rest* with *rTMS*, and *Rest* with *Sham*. Each of these four combinations was tested in a separate session, with only one session tested per day. As such, participants had four appointments per experiment, with the order of the sessions counterbalanced across participants. The third factor was Gabor location with the three levels of *Left*, *Right*, and *Bilateral* presentation.

We applied 30 trains of 20 TMS pulses, with each train lasting 2 seconds. The pulses were delivered at 60% of the maximal output of a Magstim Super Rapid rTMS device (Magstim, Whitland, UK) with a 70mm figure-of-eight coil. This stimulation intensity and duration was chosen in order to stay as close as possible to the parameters used in Jin and Hilgetag [1] and Hilgetag et al. [14].

After each train of pulses, participants either performed six trials of the visual detection task (*Task* condition) or rested passively (*Rest* condition) with the inter-train-interval set to 18 seconds. Stimulation was delivered over the intraparietal sulcus of the posterior parietal cortex, at a location which corresponds to the P4 position of the 10–20 EEG reference system [27]. During the *Test* phase, stimulation type was always *Sham* and it was applied at the same physical location as in the *Stimulation* phase by rotating the coil 90 degrees onto its narrow side.

Each phase contained 180 trials in total (i.e., 60 randomized trials for each of the tested Gabor locations) and took 10 minutes to complete, leading to 360 trials per appointment (i.e., a total of 1440 trials per experiment). Each appointment started with 126 trials (approximately 7 minutes) of behavioral practice on the visual detection task; the practice data were not included in the final data analysis.

### Data analysis

First, we computed the response accuracy (RA), that is, the number of trials in which participants responded correctly to the Gabor presentation by pressing a key on the keyboard, relative to all experimental trials. Second, we calculated the conditional response accuracy (CRA) by dividing the number of correct responses by the total number of responses. This gave us an accuracy measure that was independent of general response bias to one half of the visual field. Third, to be able to directly compare performance after stimulation with *Task* to the performance after stimulation during *Rest*, we corrected the participants’ performance after stimulation during *Rest* with regard to the time spent on task (CORR). We derived this measure by subtracting the observed performance decay in the offline phase in the *Task* condition from the performance observed after stimulation during *Rest* for both the *rTMS* and the *Sham* conditions. In order to assess any extinction-like effects of the *rTMS* stimulation, we also computed the absolute number of erroneous unilateral responses to bilateral Gabors (ERR). See Table 1 for the formulas and response measures, Table 2 for the corrections and Table 3 for the erroneous unilateral responses to bilateral Gabors.

**Table 1:** Response measures.

**Dependent measure**	**Formula**
Response accuracy (RA)	Number of correct responses / Number of trials
Response omissions (RO)	Number of omitted responses / Number of trials
Corrected RA (CRA)	Number of correct responses / Number of responses
Corrected CRA (CORR)	Rest CRA—(Task CRA—Task CRA stimulation phase)
Erroneous unilateral responses to bilateral Gabors (ERR)	Unilateral responses / Responses to bilateral trials
Reaction time (RT)	Median reaction times (RT) for correct responses

Dependent measures analyzed in the study.

**Table 2:** Correction for time-on-task.

	**rTMS**			**Sham**		
	**L**	**R**	**B**	**L**	**R**	**B**
Stimulation phase	98.8%	96.2%	85.5%	97.3%	97.4%	73.1%
Test phase	97.9%	96.9%	73.6%	96.8%	96.9%	65.1%
Correction factor	0.9%	- 0.7%	11.9%	0.6%	0.6%	8.0%

Conditional response accuracy (CRA) for the stimulation and the test phase. The last row contains the difference between the performance in the stimulation and the test phase, that is, the correction factor for time-on-task. L, R and B denote left, right and bilateral Gabors.

**Table 3:** Erroneous unilateral responses to bilateral Gabors.

	**Task**				**Rest**			
	**rTMS**		**Sham**		**rTMS**		**Sham**	
	**L**	**R**	**L**	**R**	**L**	**R**	**L**	**R**
stimulation phase	3.9	10.6	7.7	19.3	-	-	-	-
test phase uncorrected	8.9	17.4	12.8	22.2	9.8	10.5	6.3	6.7
correction factor	-	-	-	-	5.1	6.9	5.1	2.9
test phase corrected	8.9	17.4	12.8	22.2	14.9	17.4	11.4	9.5

## Results

In the following sections, we first present the data as they were collected and show that there were two effects in the data, a response bias to the right side and a time-on-task effect, that needed to be corrected for. We present the analyses of the corrected data in the subsection ‘Corrected CRA (CORR)’.

### Response accuracy (RA)

An ANOVA of the response accuracy (RA, see Table 1) with the factors stimulation type (*rTMS*, *Sham*), Gabor location (*Left*, *Right*, *Bilateral*) and condition (*Rest*, *Task*) showed that the RA was higher after *Rest* than after *Task* (82.3% vs. 71.3%, F(1,11) = 25.788, *p* < 0.001, $\eta_{p}^{2}=0.701$). This difference was expected, since participants were performing the detection task for the second time in the current experimental session in the *Task* condition and for the first time in the *Rest* condition. This time-on-task effect was also reflected in the interaction of stimulation type and condition (F(1,11) = 7.585, *p* = 0.019, $\eta_{p}^{2}=0.408$).

We therefore decided to control for time-on-task before attempting to interpret the accuracy results. Before doing so, however, we looked at the trials in which the participants did not respond.

### Response omissions (RO)

An analogue ANOVA of the response omissions (ROs, see Table 1) with the same factors revealed that participants omitted more responses when they had been performing the *Task* during stimulation rather than after *Rest* (F(1,11) = 18.538, *p* = 0.001, $\eta_{p}^{2}=0.628$). Again, we decided to correct for this time-on-task effect in the main analysis.

The ANOVA also revealed that participants omitted responses on 22.3% of all trials when the Gabor was presented in the left hemifield, 13.8% when the Gabor was presented in the right hemifield and 7% of trials with bilateral Gabors (F(2,22) = 24.536, *p* < 0.001, $\eta_{p}^{2}=0.690$). The smaller percentage of ROs on trials with bilateral Gabors was likely due to participants’ responding to one instead of both Gabors, which was an error and not an omission.

The difference in ROs between trials with left and right Gabors was significant (t(11) = 3.151, *p* = 0.0092). Since we applied sham stimulation over the right hemisphere during the test phase, it is likely that participants were cued to the right side and thus responded less to Gabors presented in their left hemifield. The interpretation of this difference as a cueing effect rather than as a stimulation-induced effect was supported by the lack of an interaction between Gabor location and stimulation type (F(2,22) = 0.355, *p* = 0.705, $\eta_{p}^{2}=0.031$). Hence, we decided to additionally correct for this response bias in the main analysis of participant’s response accuracy in addition to correcting for time-on-task.

The interaction of the main effects was significant (Condition x Gabor location, F(2,22) = 3.533, *p* = 0.047, $\eta_{p}^{2}=0.243$). No other main effects or interactions were significant (all *Fs* < 2.3, all *ps* > 0.15).

### Correcting for response bias and time-on-task

We corrected for the observed response bias to the right side by dividing the number of correct responses by the number of button presses in the respective combination of stimulation type, Gabor location and condition (conditional response accuracy (CRA), see Table 1).

To be able to directly compare performance after stimulation with *Task* to the performance after stimulation during *Rest*, we corrected the participants’ performance after stimulation during *Rest* with regard to the time spent on task. We derived this measure by subtracting the observed performance decay from the stimulation phase to the test phase in the *Task* condition from the performance observed after stimulation during *Rest* for both the *rTMS* and the *Sham* conditions (corrected CRA (CORR), see Table 1). Table 2 shows the CRA during and after stimulation with *Task*, as well as the resulting correction factor.

An ANOVA of the CRAs with the factors phase (*Stimulation*, *Test*), stimulation type (*rTMS*, *Sham*) and Gabor location (*Left*, *Right*, *Bilateral*) confirmed that the observed performance decay was significant (main effect of phase, Stimulation: 91.4%, Test: 87.9%, F(1,11) = 12.485, *p* = 0.005, $\eta_{p}^{2}=0.532$).

### Corrected CRA (CORR)

**Fig 2:**
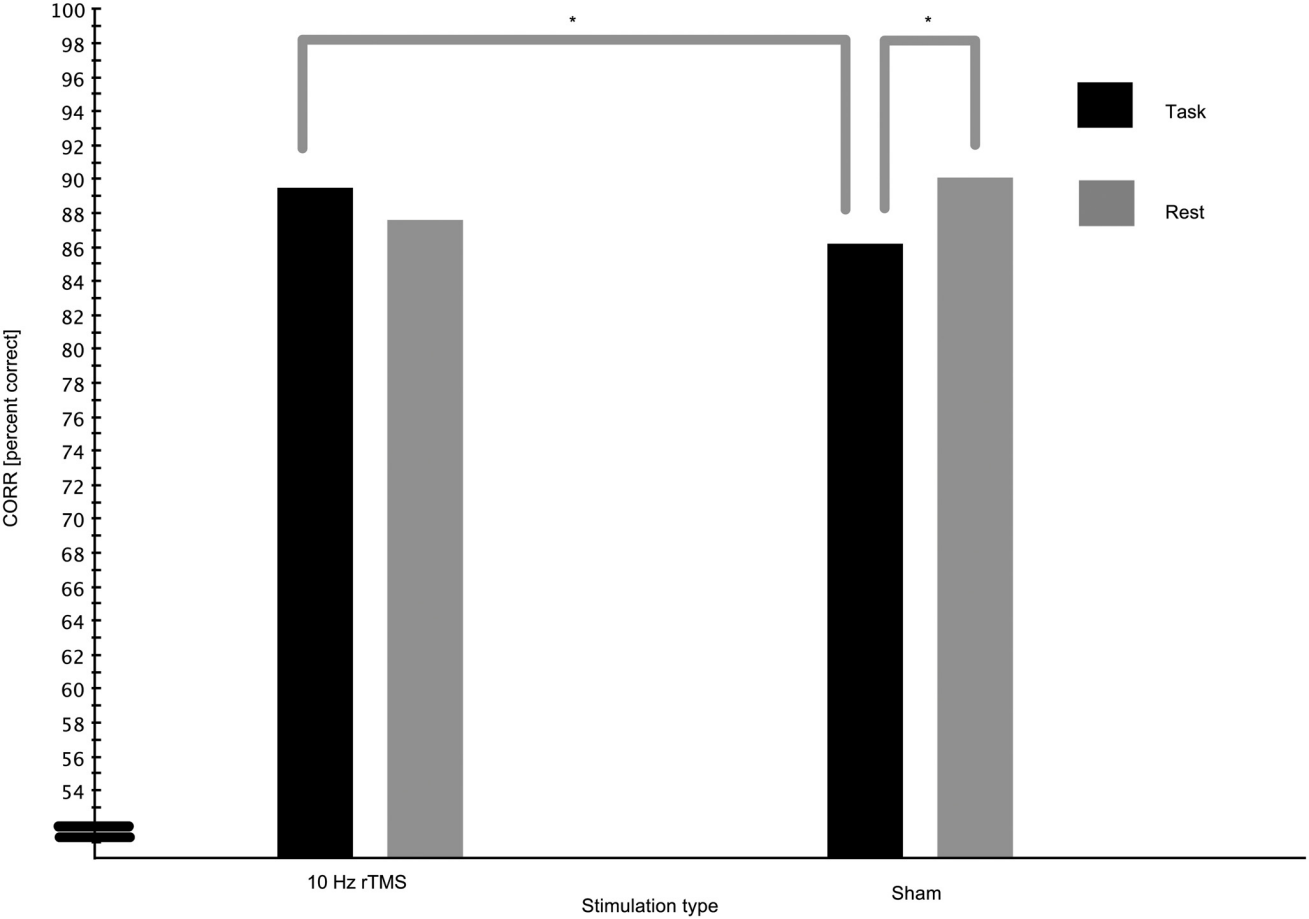
Corrected conditional response accuracies (CORR) after 10 Hz rTMS. Stars indicate significant differences after correcting for time on task as indicated by one-tailed paired t-tests with *p* < 0.05 after correcting for multiple comparisons.

Fig 2 depicts the results of the main analysis (see also Table 4). After correcting for time-on-task and the response bias to the right, an ANOVA on the corrected CRA (CORR, see Table 1) with the factors stimulation type (*rTMS*, *Sham*), Gabor location (*Left*, *Right*, *Bilateral*) and condition (*Rest*, *Task*) revealed an interaction of stimulation type and condition (F(1,11) = 12.832, *p* = 0.004, $\eta_{p}^{2}=0.538$).

**Table 4:** Results.

	**Task**						**Rest**					
	**rTMS**			**Sham**			**rTMS**			**Sham**		
	**L**	**R**	**B**	**L**	**R**	**B**	**L**	**R**	**B**	**L**	**R**	**B**
RA	71.5	82.4	69.6	67.8	77.5	58.8	80.1	86.1	76.0	82.9	86.3	82.5
CRA	97.9	96.9	73.6	96.8	96.9	65.1	98.6	96.6	79.7	96.8	95.7	87.1
CORR	97.9	96.9	73.6	96.8	96.9	65.1	97.7	97.3	67.8	96.2	95.2	79.1
RO	26.9	14.9	6.8	29.6	19.7	10.7	18.6	10.8	5.0	14.0	9.6	5.4
ERR	8.9	17.4	-	12.8	22.2	-	14.9	17.4	-	11.4	9.5	-
RT	443	447	439	457	457	442	420	424	420	420	424	420

Summary of percent correct response accuracy (RA), conditional response accuracy (CRA), response omissions (RO), erroneous unilateral responses to bilateral Gabors after correcting for time-on-task (ERR) and performance corrected for the time spent of task (CORR). Note that for the task, CORR is identical to CRA, since the time-on-task correction was applied only to the rest data. Reaction times (RT) are listed in milliseconds (ms).

Performance after *rTMS* with *Task* did not differ significantly from performance after *rTMS* during *Rest* (89.5% vs. 87.6%, t(11) = 1.7140, *p* = 0.0573, one-tailed t-test). There was also no performance difference between performance after *rTMS* during *Rest* and *Sham* stimulation during *Rest* (87.6% vs. 90.1%, t(11) = 1.1413, *p* > 0.1). However, performance after *Sham* stimulation was better when participants rested during stimulation (*Rest*) than when they were performing the *Task* (90.1% vs. 86,2%, t(11) = 2.2826, *p* = 0.0082, one-tailed t-test). Performance after stimulation with *rTMS* while performing the *Task* was better than after *Sham* stimulation with *Task* (89.5% vs. 86.2%, t(11) = 2.7536, *p* = 0.0094, one-tailed t-test, all tests Bonferroni corrected, significance level *p* = 0.0125).

A significant three-way interaction of Gabor location, stimulation type and condition (F(2,22) = 14.637, *p* < 0.001, $\eta_{p}^{2}=0.571$) showed that the interaction reported above differed between locations. When the Gabor was presented in the left hemifield, there was a small non significant trend towards better performance after rTMS than after sham stimulation, irrespective of condition (97.8% vs. 96.5%, t(11) = 1.6824, *p* = 0.0603, one-tailed t-test). Performance did not vary with stimulation type or condition, when the Gabor was presented on the right side of the screen (all *ts* < 0.91).

When the Gabors were presented bilaterally, there was a non-significant trend to better performance after *rTMS* with *Task* than after stimulation with *Task* durig *Rest* (73.6% vs. 67.8%, t(11) = 1.8505, *p* = 0.0457, one-tailed paired t-test). Performance was better after *rTMS* with *Task* than after *Sham* stimulation with *Task* (73.6% vs. 65.1%, t(11) = 2.6384, *p* = 0.0115, one-tailed t-test). However, there was also a trend towards a deteriorated performance after *rTMS* during *Rest* than after *Sham* during *Rest* (79.1% vs. 67.8%, t(11) = 1.799, *p* = 0.0497, one-tailed t-test, all tests Bonferrroni corrected, significance level *p* = 0.0125). The results for bilaterally presented Gabors thus drove the interaction reported above and depicted in Fig 2.

The main effect of Gabor location (left: 97.1%, right: 96.5%, bilateral: 71.4%, F(2,22) = 42.564, *p* < 0.001, $\eta_{p}^{2}=0.795$) and the interaction of Gabor location with condition (F(2,22) = 6.674, *p* = 0.005, $\eta_{p}^{2}=0.378$) were also significant. No other main effects or interactions were significant (all *Fs* < 1.1, all *ps* > 0.3).

### Erroneous unilateral responses to bilateral Gabors (ERR)

We now return to the trials in which participants’ mistook two bilaterally presented Gabors for a unilaterally presented Gabor. Table 3 lists the percentage of ERRs for each condition and phase, as well as the correction factors applied to the data.

Since we found a performance decay from the *Stimulation* to the *Test* phase (10.4% vs. 15.3%, F(1,11) = 15.378, *p* = 0.002, $\eta_{p}^{2}=0.583$) also for the erroneous unilateral responses to bilateral Gabors, we corrected for this time-on-task effect in the same way as for the RAs reported above.

An ANOVA with the factors stimulation type (*rTMS*, *Sham*), response side (*Left*, *Right*) and condition (*Task*, *Rest*) showed that participants made on average 2% more ERRs after stimulation with *Task* than after stimulation during *Rest* (15.3% vs. 13.3%, F(1,11) = 4.995, *p* = 0.047, $\eta_{p}^{2}=0.321$). The effect of condition interacted with stimulation type (F(1,11) = 17.135, *p* = 0.002, $\eta_{p}^{2}=0.609$) and with response side (F(1,11) = 8.384, *p* = 0.015, $\eta_{p}^{2}=0.433$).

Similar to the analysis of CORRs, there was a trend towards better performance after *rTMS* with *Task* than after *rTMS* during *Rest* (13.2% vs. 16.1%, t(11) = 1.8505, *p* = 0.0456, one-tailed t-test). Performance after *rTMS* with *Task* was better than after *Sham* with *Task* (13.19% vs. 17.47%, t(11) = 2.6384, *p* = 0.0115, one-tailed t-test). Furthermore, participants made less erroneous unilateral responses to the left side after *rTMS* with *Task* than after *rTMS* during *Rest* (8.9% vs. 14.9%, t(11) = 3.0050, *p* = 0.012), while the erroneous responses to the right side did not differ (*rTMS/Task*: 17.4%, *rTMS/Rest*: 17.4%, t(11) = 0.0142, *p* = 0.9889).

### Reaction time (RT)

An ANOVA with the factors stimulation type (*rTMS*, *Sham*), phase (*Stimulation*, *Test*) and Gabor location (*Left*, *Right*, *Bilateral*) of the stimulation phase showed that the participants’ responses were slower in the test phase than in the stimulation phase (test phase: 528ms, stimulation phase: 477ms, F(1,11) = 28.075, *p* < 0.001, $\eta_{p}^{2}=0.718$). After correcting for time-on-task, an ANOVA with the factors stimulation type (*rTMS*, *Sham*), Gabor location (*Left*, *Right*) and condition (*Task*, *Rest*) did not reveal any effects on RTs (all *Fs* < 3.7, all *ps* > 0.08, see Tables 1 and 4).

### Stimulation phase

*Response accuracy*. We analyzed the participants’ performance during the stimulation phase with an ANOVA of the RAs with the factors stimulation type (*rTMS*, *Sham*) and Gabor location (*Left*, *Right*, *Bilateral*) did not show a significant difference between performance with rTMS and performance with sham stimulation (rTMS: 83.8% correct, sham: 76.1% correct, F(1,11) = 3.236, *p* = 0.1, $\eta_{p}^{2}=0.227$). The interaction of stimulation type and Gabor location was also not significant (F(2,22) = 2.249, *p* = 0.129, $\eta_{p}^{2}=0.170$). The main effect of Gabor location was significant (Left: 78.6%, Right: 85.9%, Bilateral: 75.3%, F(2,22) = 6.631, *p* = 0.019, $\eta_{p}^{2}=0.376$).

*Response omissions*. An ANOVA with the factors stimulation type (*rTMS*, *Sham*) and Gabor location (*Left*, *Right*, *Bilateral*) showed that ROs differed for the three Gabor locations (main effect of Gabor location, F(2,22) = 9.329, *p* = 0.006). Participants omitted 19.79% of the Gabors presented on the left, 11.25% of the Gabors presented on the right and 5.9% of bilaterally presented Gabors. No other main effects or interactions were significant (all *Fs* < 1.3, all *ps* > 0.2).

*Erroneous unilateral responses to bilateral Gabors*. A similar ANOVA with the factors stimulation type (*rTMS*, *sham*) and response side (*Left*, *Right*) on the ERRs showed that participants made more ERRs to the right side than to the left side (Left: 5.8%, Right: 14.9%, main effect of response side, F(1,11) = 6.933, *p* = 0.023, $\eta_{p}^{2}=0.387$). Participants made less ERRs with rTMS (7.2%) than with sham stimulation (13.5%, main effect of stimulation type, F(1,11) = 5.360, *p* = 0.041, $\eta_{p}^{2}=0.328$).

*Reaction time*. An ANOVA with the factors stimulation type and Gabor location on the RTs did not reveal any main effects or interactions (all *Fs* < 2.9, all *ps* > 0.08).

## Discussion

The aim of the present study was to investigate whether the effects of high frequency rTMS applied to the right PPC during passive rest differ from the effects of high frequency rTMS applied to the same location while participants performed a visual detection task that was also used in the subsequent test phase. We found several indications that visual detection might be better after rTMS with task than after rTMS during passive rest, however most of the direct comparisons just missed statistical significance.

Overall, we found a trend towards better visual detection after rTMS with task than after rTMS during rest, which was supported by a second trend towards better detection in the left half of the visual field after rTMS with task than after rTMS during rest. Detection performance in the right half of the visual field did not differ after rTMS with task and rTMS during rest. A third trend indicated that performance for bilateral Gabors was better after rTMS with task than after rTMS during rest, which was supported by the fact that participants made significantly less erroneous unilateral responses to bilateral Gabors after rTMS with task than after rTMS during rest. This lead to a significantly more pronounced bias to the right side of the visual field whenever participants made an erroneous unilateral response.

Had all the statistical comparisons been significant, these results would have exactly matched our expectations. High frequency rTMS in combination with a task, but not after stimulation during rest, would have lead to mirrored effects of low frequency rTMS by improving the detection of visual stimuli in the hemifield contralateral to stimulation. However, the results of the present experiment do not allow us to conclude that this was indeed the case. Thus, we cannot reject the null hypothesis that high frequency rTMS with task and high frequency rTMS during rest equally affect performance.

Nevertheless, we consistently found that visual detection after 10 Hz rTMS with task was overall better than after sham stimulation applied concurrently with the same task. This effect might be due to two factors. First, the fact that we applied rTMS in a separate stimulation phase, that is, before the assessment of the participants performance in the test phase. And second, that we applied rTMS at a frequency of 10 Hz.

It is well known that the effects of rTMS on task performance depend on when they are applied relative to the task. High frequency stimulation applied for several minutes before the start of the experiment appears to enhance performance, whereas stimulation during task execution tends to lead to a performance deterioration, see e.g. [28, 29]. Romei and Pascual-Leone, for example, applied 5 pulses of rTMS at 10 Hz to the PPC while participants were performing a visual detection task [30]. In contrast to our study, they did not stimulate before the experiment, but at the beginning of the trial, just prior to target onset. The authors did not observe an increase, but a decrease in detection performance in the hemifield contralateral to stimulation.

In contrast to Romei and Pascual-Leone [30], Kim, Min, Ko, Park, Jang and Lee [31] stimulated the PPC for 20 minutes with 10 Hz before their participants performed a line bisection task. They found that performance with stimuli that were assessing attentional processing in the hemifield contralateral to stimulation was enhanced relative to a pre-stimulation baseline. In the present study, we achieved a similar performance enhancement by applying 10 Hz rTMS in a separate stimulation phase for a visual detection task.

Moreover, it has been shown that the effects of rTMS can be frequency-specific. For example, Chanes, Quentin, Tallon-Baudry and Valero-Cabre [32] found an improved detection sensitivity for stimuli in both visual hemifields after short (4 pulses) trains of 30 Hz stimulation of the right frontal eye field immediately before target onset, but not after 50 Hz stimulation or after irregular pulses. This specificity of stimulation frequency might explain, why Jin and Hilgetag [1] observed a decrease in detection performance after 20 Hz stimulation, whereas we saw an improvement after 10 Hz stimulation.
